# Supplementary figures and images for: Using Entamoeba muris To Model Fecal-Oral Transmission of Entamoeba in Mice
Source: mBio. 2023 Feb 6;14(1):e03008-22. doi: 10.1128/mbio.03008-22 (PMC9973306; doi:10.1128/mbio.03008-22)

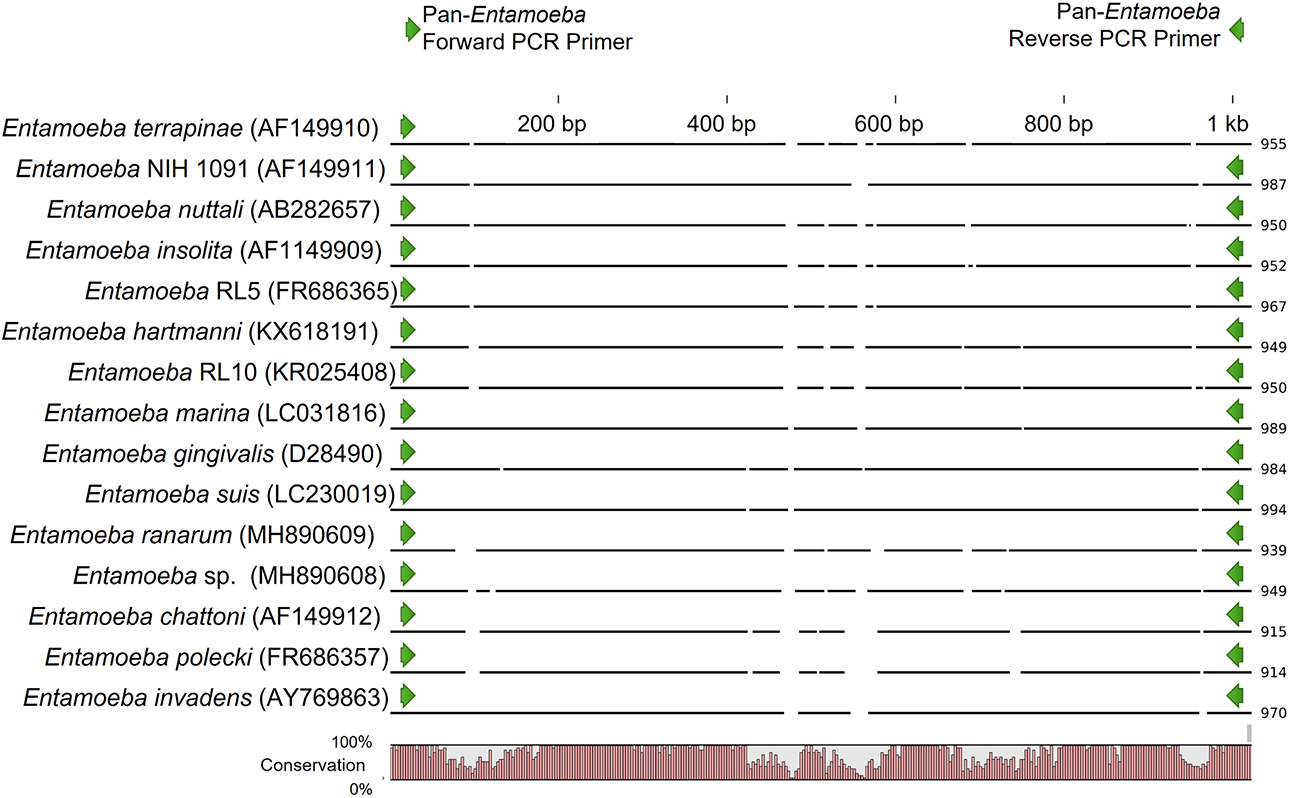

Supplement: FIG S1 [file mbio.03008-22-s0001.tif]

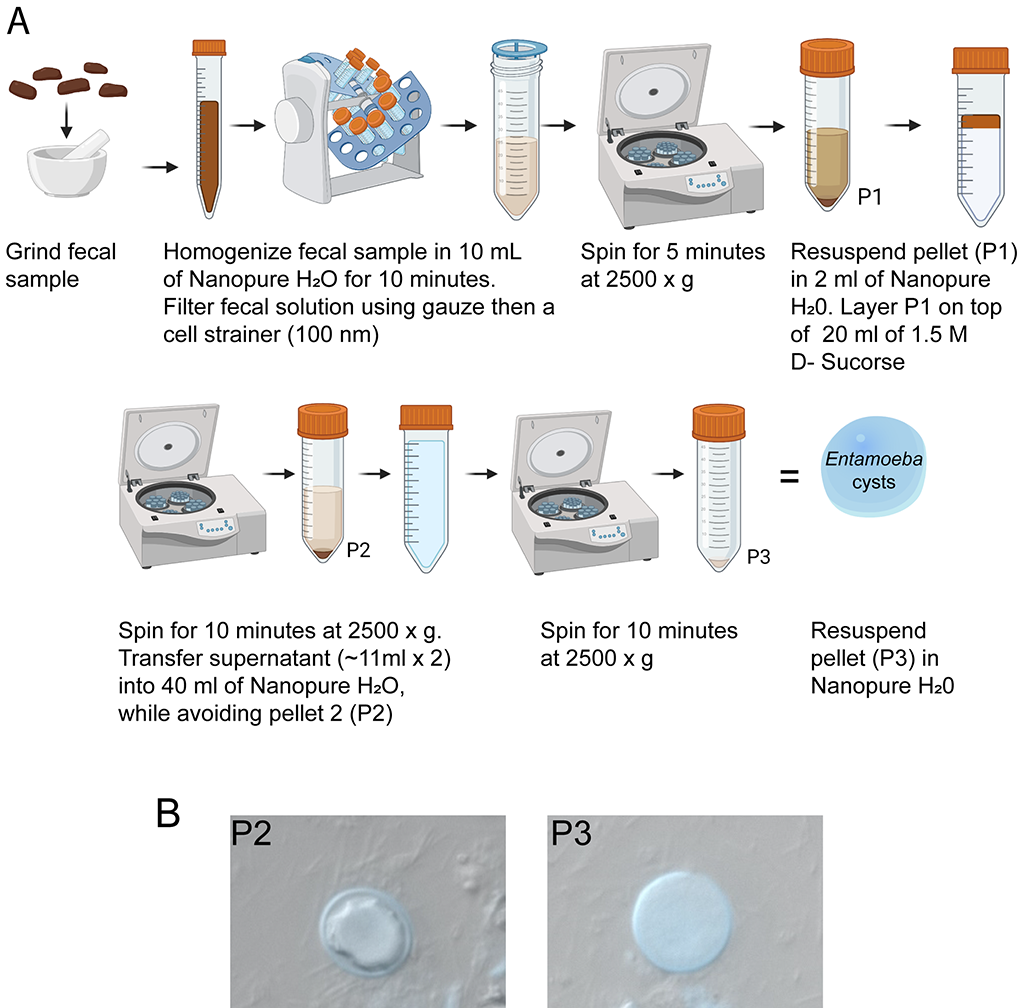

Supplement: FIG S2 [file mbio.03008-22-s0002.tif]

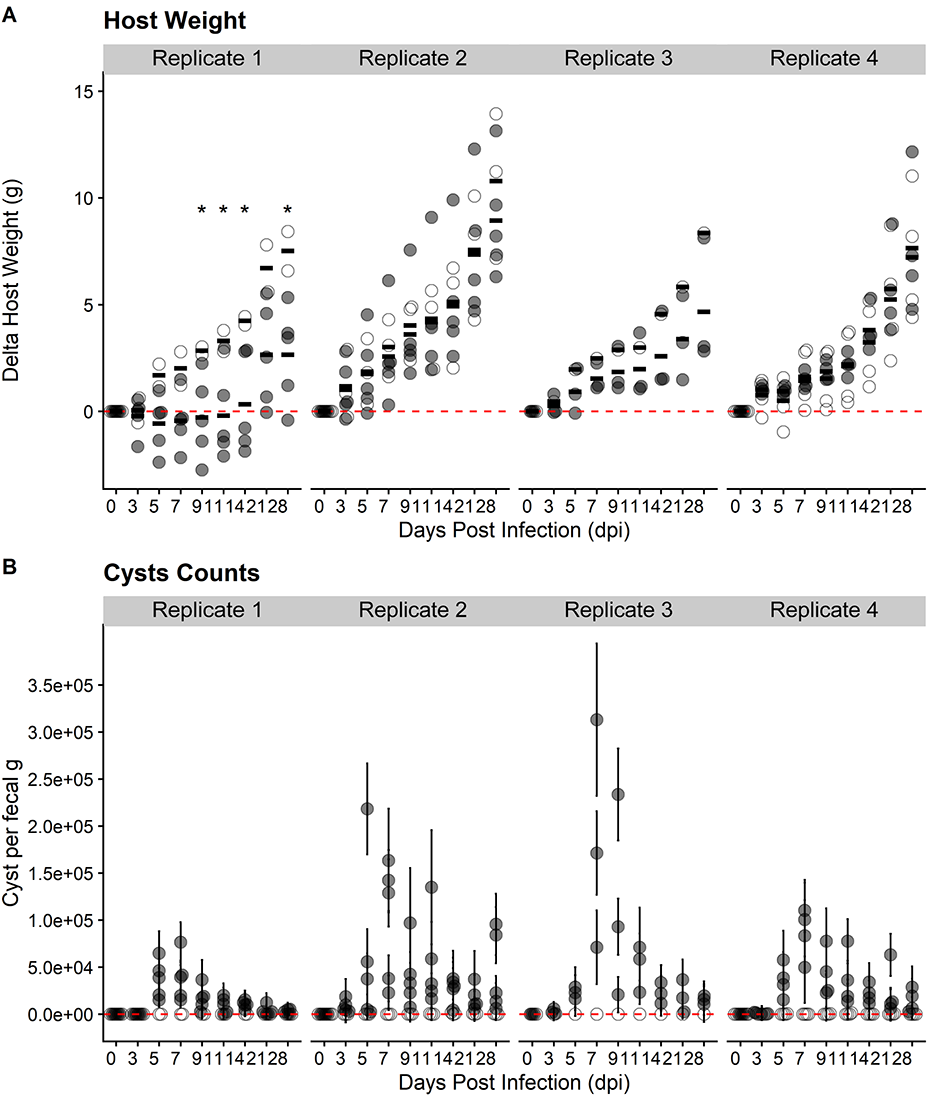

Supplement: FIG S3 [file mbio.03008-22-s0003.tif]

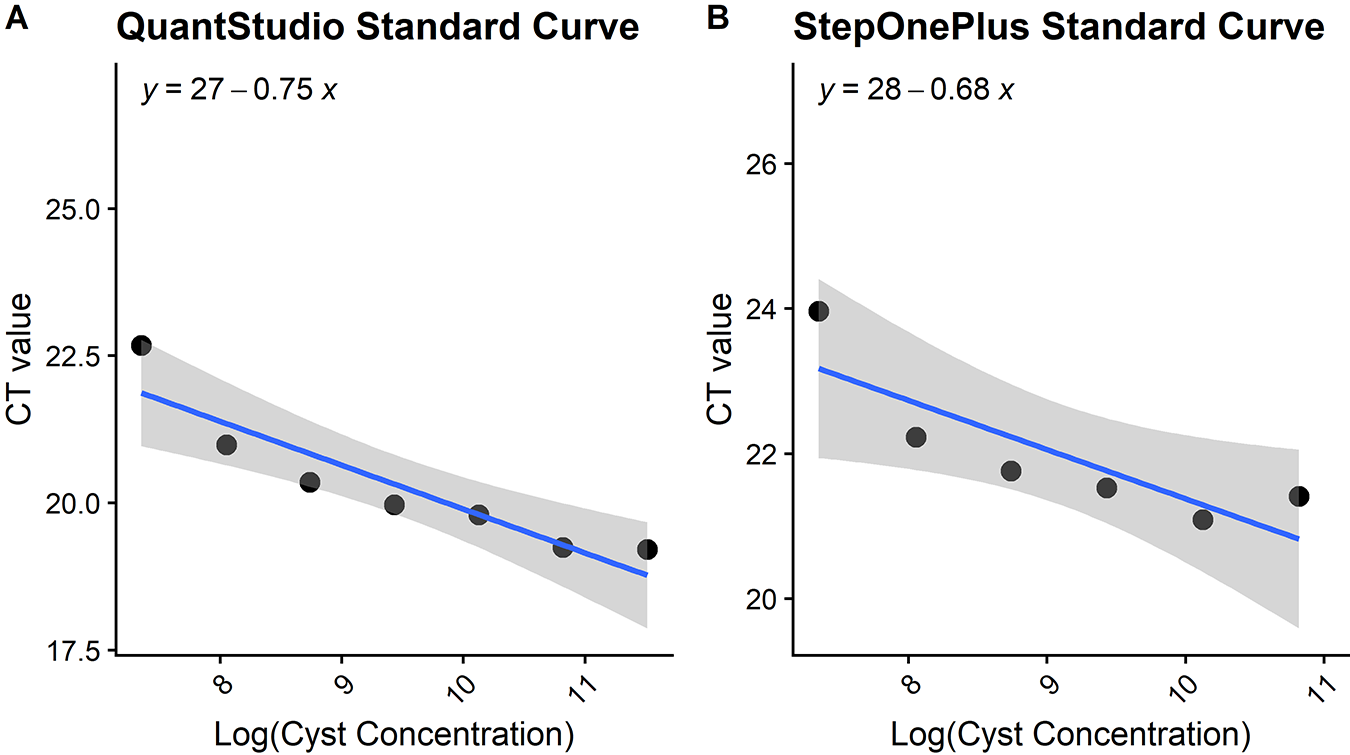

Supplement: FIG S4 [file mbio.03008-22-s0004.tif]

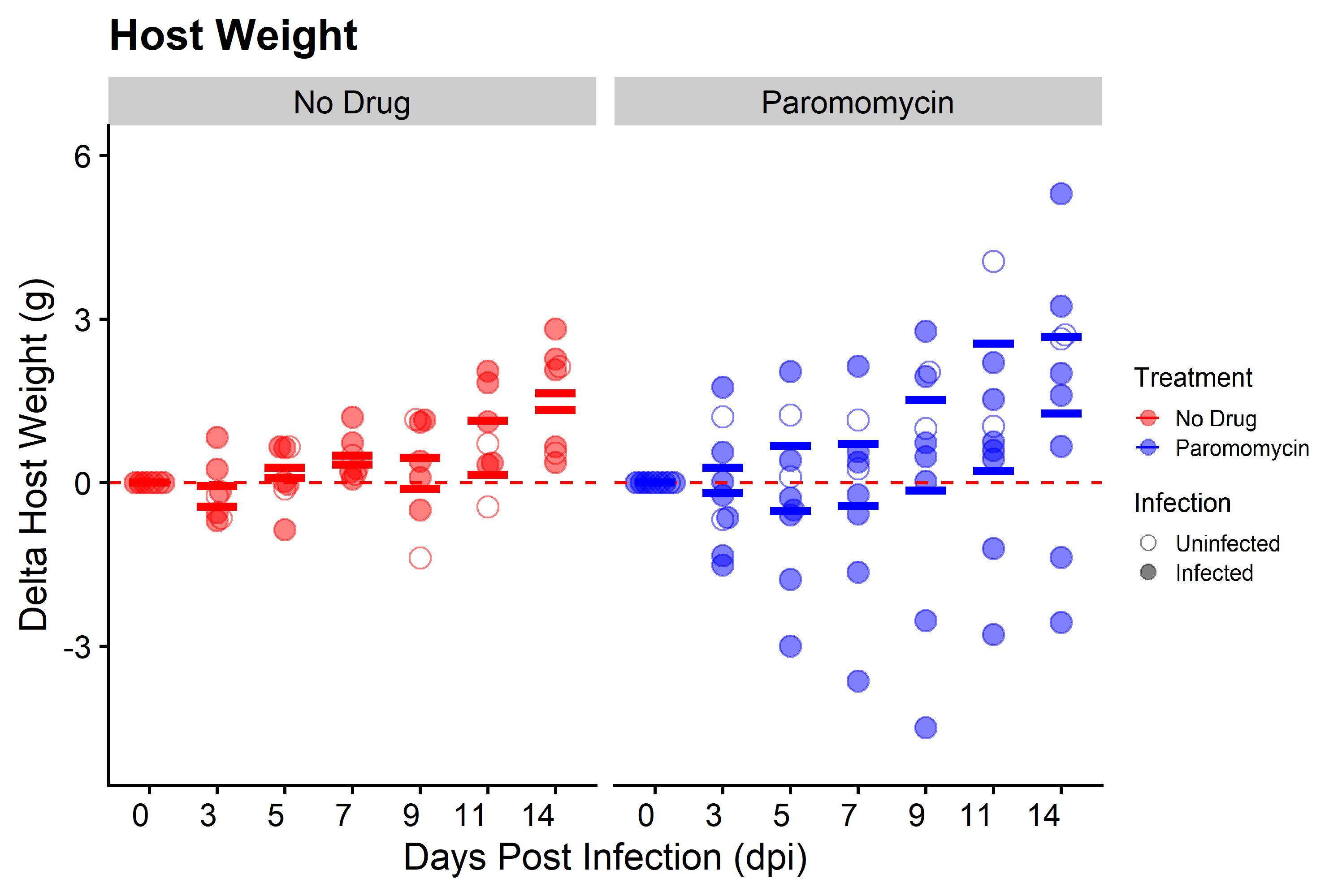

Supplement: FIG S5 [file mbio.03008-22-s0005.tif]

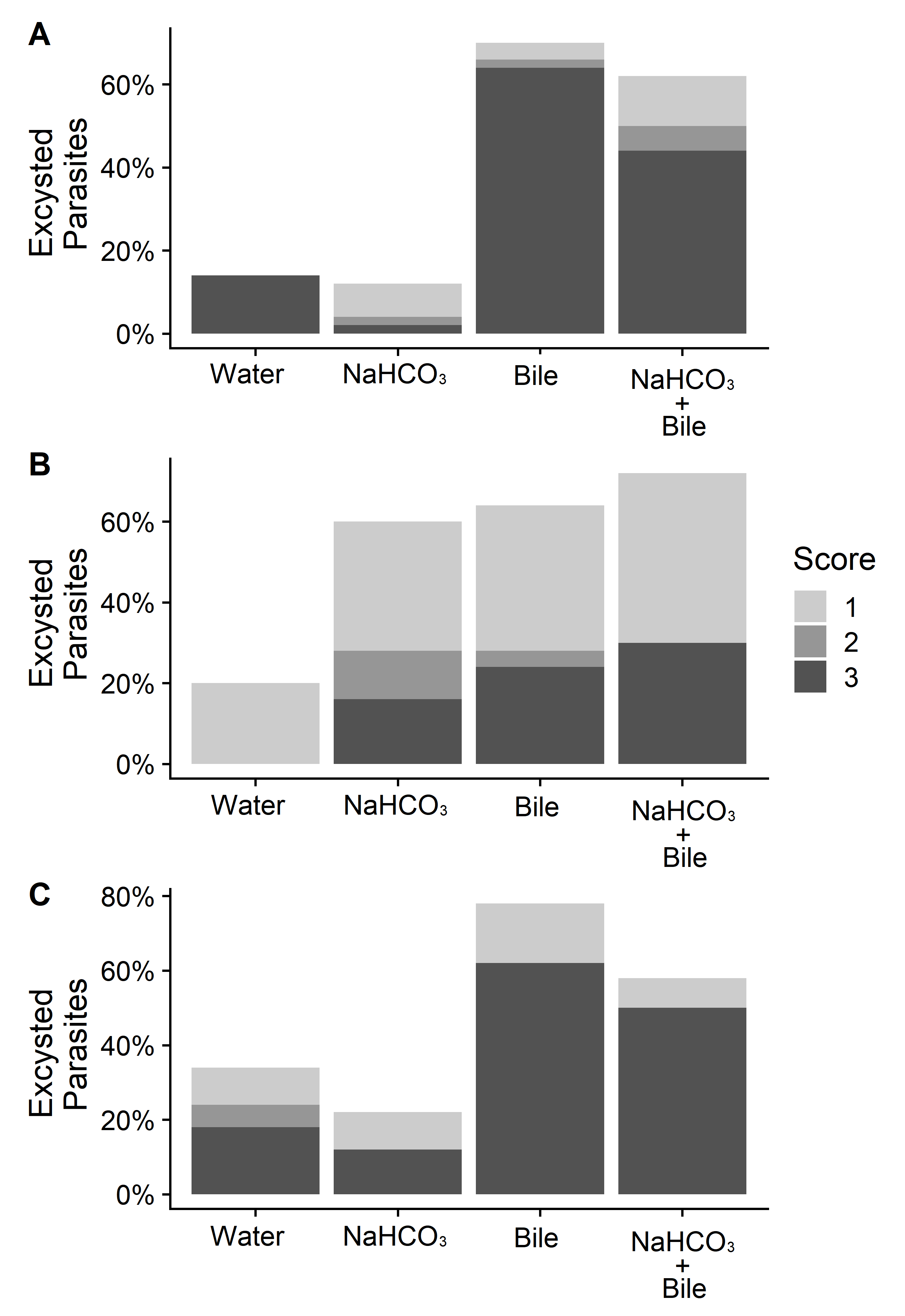

Supplement: FIG S6 [file mbio.03008-22-s0006.tif]
