# Supplementary material for: Using Entamoeba muris To Model Fecal-Oral Transmission of Entamoeba in Mice
Source: mBio. 2023 Feb 6;14(1):e03008-22. doi: 10.1128/mbio.03008-22 (PMC9973306; doi:10.1128/mbio.03008-22)
Supplement: TABLE S1 [file mbio.03008-22-s0007.docx]

| Institution  Location | B6  Samples | % Positive  (40-cycle PCR) | % Positive  (Nested PCR) |
| --- | --- | --- | --- |
| Arizona | N= 4 | 0% | 25% |
| California | N= 2 | 100% | 100% |
| Pennsylvania | N= 2 | 0% | 0 % |
| Texas | N=1 | 0% | 0% |
| Vermont | N=10 | 0% | 0% |
