# Supplementary material for: Using Entamoeba muris To Model Fecal-Oral Transmission of Entamoeba in Mice
Source: mBio. 2023 Feb 6;14(1):e03008-22. doi: 10.1128/mbio.03008-22 (PMC9973306; doi:10.1128/mbio.03008-22)
Supplement: TABLE S2 [file mbio.03008-22-s0008.docx]

|  | *Entamoeba*  *coli* | *Entamoeba*  RL7 | *Entamoeba muris* | Consensus sequence |
| --- | --- | --- | --- | --- |
| *Entamoeba coli* |  | 77.9 | 77.6 | 76.1 |
| *Entamoeba* RL7 | 51 |  | 82.6 | 81.7 |
| *Entamoeba muris* | 44 | 37 |  | 91.6 |
| Consensus sequence | 49 | 42 | 9 |  |
